# After-School Extracurricular Activities Participation and Depressive Symptoms in Chinese Early Adolescents: Moderating Effect of Gender and Family Economic Status

**DOI:** 10.3390/ijerph19074231

**Published:** 2022-04-01

**Authors:** Yangu Pan, Di Zhou, Daniel T. L. Shek

**Affiliations:** 1 Research Institute of Social Development, Southwestern University of Finance and Economics, Chengdu 611130, China; panyg@swufe.edu.cn (Y.P.); zhoudi936@163.com (D.Z.); 2 Department of Applied Social Sciences, The Hong Kong Polytechnic University, Hong Kong 999077, China

**Keywords:** extracurricular activity, depressive symptoms, gender, family economic status, mainland China

## Abstract

Although Western studies showed that participation in extracurricular activities was intimately linked to adolescents’ psychological adjustment, very few studies have addressed this issue among early adolescents in China. Based on a nationally representative sample of 9672 Chinese junior high school students (*M*_age_ = 14.54 years, *SD* = 0.70 years), this study investigated the relationship between participation in different extracurricular activities and depressive symptoms among Chinese early adolescents, and the moderating role of gender and family economic status. Results indicated that time spent completing homework, attending extracurricular tutoring, and playing online games after school was positively related to students’ depressive symptoms, whereas time spent on participating in physical exercise was negatively associated with students’ depressive symptoms. Besides, the relationships between after-school activities participation and student depressive symptoms were moderated by gender and family economic status. The theoretical and practical implications for the arrangement of after-school activities for Chinese early adolescents are discussed.

## 1. Introduction

Depression is a serious mental health problem that has detrimental effects on adolescent psychosocial functioning [1]. Early adolescents are vulnerable to depression [2,3] because early adolescence is an important transition period from childhood to adolescence during which adolescents face challenges and stressors arising from different psychosocial domains [4,5]. According to the Report on National Mental Health Development in China (2019–2020), 24.6% of adolescents suffered from depression in China in 2020, with 7.4% and 17.2% with major depression and mild depression, respectively [6]. Moreover, a meta-analysis showed that the prevalence of depressive symptoms among adolescents in middle schools in mainland China was 24.3% [7]. As depressive symptoms have adverse impacts on early adolescents’ psychosocial adjustment, it is essential to identify related protective and risk factors to inform prevention and intervention directions.

Participation in extracurricular activities, such as physical exercise, could protect adolescents from depressive symptoms [8]. In the United States, after-school activities are a very important part of the public service system, which could help students effectively use their after-school time and prevent students from having behavioral problems [9]. In China, students participate in many kinds of after-school activities, including doing homework, extracurricular tutoring, attending interest classes, having physical exercise, watching TV, and surfing the Internet as well as playing online games [10]. However, very few studies have explored the relationship between participation in different extracurricular activities and student psychological adjustment. Moreover, few studies have examined moderators involved in the relationships between after-school activities and psychological well-being. Furthermore, existing studies have seldom employed nationally representative samples to examine this issue, including in China. Therefore, this study investigated the relationships between participation in extracurricular activities and depressive symptoms among a nationally representative sample of junior high school students in China, as well as the related moderators.

### 1.1. Types of Extracurricular Activities in China

In China, the Central Government clearly requires schools to enrich students’ extracurricular activities, reduce their academic burden, and comprehensively promote quality education, as well as to encourage students to participate in society and gain practical experience [11]. However, influenced by an examination-oriented education culture, Chinese students’ extracurricular activities are characterized by “schoolization”. In reality, the time spent on schoolwork accounts for a large proportion of Chinese students’ after-school time, and extracurricular tutoring is increasing [12,13]. Cross-culturally speaking, Hu [14] investigated extracurricular activities among students from China, Japan, and the United States, and found that Chinese students spent the most time on study and the least time on practical activities in their free time.

Studies showed that extracurricular activities among primary and middle school students in China mainly include learning activities and recreational activities. Wu et al. [15] investigated 5976 teenagers’ extracurricular learning in Beijing and found that extracurricular learning was mainly an extension of school learning and that participation in extracurricular tutoring (tutorial classes, family tutoring) was common. Xie and Jia [12] also reported that 45.2% of students attended various interest classes that they liked or disliked (probably arranged by their parents) on weekends and holidays. Moreover, for leisure and entertainment activities, Chen and Du [16] found that 55% of students surfed the Internet during holidays and that 59% surfed the Internet every day. Besides, many primary and middle school students showed addictive symptoms in online games [6]. In addition, 65% of students spent their holidays watching TV and movies.

### 1.2. Participation in After-School Activities and Adolescent Depressive Symptoms

Regarding the relationship between participation in after-school activities and depressive symptoms, Oberle et al. [17] found that sedentary leisure activities (such as watching TV and surfing the Internet and playing video games) were positively related to adolescent depressive symptoms, while physical sports and interest class extracurricular activities were negatively associated with adolescent depressive symptoms. Other studies also found that teenagers who were addicted to the Internet or video games or spending too much time watching TV tended to have anxiety, depression, sensitivity, withdrawal, inferiority, a lack of social courage, and behavioral problems [18,19,20].

Moreover, studies have shown that spending long hours on homework was positively related to student depressive symptoms. Yeo et al. [21] surveyed 1225 adolescents from eight secondary schools in Singapore and found that those who spent long hours doing homework or studying showed more depressive symptoms and that those who spent five hours a day doing homework on weekends showed anhedonia and anxiety symptoms. With regard to the relationship between tutoring and depressive symptoms, Kuan [22] found that although tutoring could improve students’ academic performance, it also significantly increased their risk of depression. Dui and Li [23] found that moderate tutoring could improve children’s non-cognitive ability, but excessive tutoring reduced non-cognitive ability and increased children’s depression. According to the stress theory of depression, when individuals confront stressful life events, latent negative or depressogenic self-schemas are activated in an automatic, repetitive, unintended, and uncontrollable way [24]. This leads to specific negative cognitions (automatic thoughts), including negative views of oneself, resulting in sadness and other depressive symptoms [24,25]. As Chinese parents generally believe that adolescence is the golden stage of life, learning is regarded as the main task of teenagers, resulting in increased academic pressure, which constitutes an important stressful life event for teenagers [26]. Fan et al. [27] investigated depressive disorder in middle school students, and found that learning burden, failure or unsatisfactory examination, and pressure of further studies were the top three stressors faced by adolescents.

On the other hand, there are studies showing that participation in physical exercise can keep individuals in a good state of mind and protect them from depressive symptoms and anxiety [28,29,30]. Physical activity is useful both in preventing chronic and degenerative diseases and for socialization, which is vital for the optimal physical and psychological development of adolescents [31,32]. Besides, regarding the relationship between participation in interest classes and adolescent depressive symptoms, studies suggested that art education might stimulate the right brain and promote the function of the left brain, which is beneficial to student mental health and harmonious interpersonal relationships [33,34].

### 1.3. Gender and Family Economic Status as Moderators

Due to gender differences in physiological and psychological development during adolescence, the associations of participation in different types of extracurricular activities with depressive symptoms may vary between boys and girls. First, compared with boys, girls showed more depressive symptoms when they faced stress [35,36]. Thus, when faced with the same academic pressure, girls may show more depression symptoms than boys. Second, due to biological sex differences and cultural expectations, boys and girls might have different interests in the use of media such as television and the Internet. Ha and Hwang [37] found gender differences in Internet addiction behaviors, with higher prevalence of Internet addiction in boys than in girls. Moreover, researchers suggested that gender might moderate the relationship between problematic Internet use and mental health. Liang et al. [38] argued that as girls spent a large amount of time chatting online and browsing social networking sites (hence leading to a reduction in social support in real life), girls might become more sensitive to a lack of interpersonal relationships. As such, problematic Internet use might be more positively associated with depressive symptoms among girls than boys. Third, with regard to the role of gender on the relationship between physical exercise and adolescent depressive symptoms, Liu [39] found that the frequency and intensity of physical exercise in boys were usually higher than in girls. Thus, relative to boys, girls could obtain higher marginal returns from physical exercise, with physical exercise having more positive effects on the mental health among girls.

In addition to gender, family economic status may moderate the association between participation in after-school extracurricular activities and depressive symptoms among early adolescents. Zhang and Wu [40] found that extra-curricular tutoring (tutorial classes, family tutoring) mainly occurs in socioeconomically middle- and high-class children. However, children from a lower class might benefit from homework, extra-curricular tutoring, and interest classes because the related activities would promote human capital, which would help them to move up the social ladder. Moreover, children with high family economic status spent more time on watching TV and surfing the Internet and playing online games than did children with low family economic status [41] probably because they have more financial resources (such as paying for the Wifi data) and less parental supervision. On the other hand, such activities may simply be “entertainment” for poor children because they usually have limited financial resource. Furthermore, studies showed that SES differences were potential correlates of physical activity among adolescents. For example, some studies found a positive relationship between families SES and participation in extra-curricular physical activity [32,42], whereas other studies found a negative relationship [43,44].

### 1.4. The Present Study

Although previous studies suggested that extra-curricular activities are related to adolescent development, there are several limitations of the scientific literature. First, previous literature on adolescent participation in after school activities tends to focus on structured after-school activities, with few studies investigating the relationship between unstructured after-school activities and adolescents’ well-being. Second, previous studies are predominantly Western studies with very few studies examining this issue in non-Western contexts such as China [45,46]. Third, the sample size in some studies was small [8]. Finally, very few studies have explored the mediators between adolescents’ participation in extracurricular activities and their development, such as gender and family economic status. Therefore, the current study aimed to examine the relationship between Chinese adolescents’ participation in after-school extracurricular activities and depressive symptoms, as well as the moderating effects of gender and family economic status. Specifically, the present study attempted to answer the following five questions.

Question 1: Are there gender differences in the participation in different kinds of extra-curricular activities and depression in early adolescents? Based on the literature, we proposed that girls would spend more time on homework [47,48], extracurricular tutoring [49,50], and interest classes [33] (Hypothesis 1a), while boys would spend more time on watching TV, surfing online and playing online games [51,52,53], and physical exercise [39,54] (Hypothesis 1b). Additionally, with reference to the literature [35,36,55], we proposed that girls would have greater depressive symptoms compared to boys (Hypothesis 1c).

Question 2: Do adolescents from different family economic status differ in their participation in different types of extra-curricular activities and depressive symptoms? Based on previous studies, we proposed that time spent on homework, extracurricular tutoring, interest classes [50,56], and physical exercise [31,32] would be higher among students with a higher family economic status (Hypothesis 2a), while time spent on watching TV and surfing online and playing online games [57] would be higher among students with a lower family economic status (Hypothesis 2b). Based on the scientific literature [58], we also proposed that students with a lower family economic status would have greater depressive symptoms relative to students with a high family economic status (Hypothesis 2c).

Question 3: Is participation in different after-school activities related to depressive symptoms among Chinese early adolescents? Based on the literature, we proposed time spent on homework [21], extracurricular tutoring [22,23], watching TV, and surfing online and playing online games [18,19,20] would be positively associated with depressive symptoms among Chinese early adolescents (Hypothesis 3a). On the other hand, time spent on interest classes [33,34] and physical exercise [28,29,30] would be negatively associated with depressive symptoms among Chinese early adolescents (Hypothesis 3b).

Question 4: Does gender moderate the relationship between participation in after-school activities and depressive symptoms among Chinese early adolescents? Based on the previous studies, we proposed that time spent on homework, extracurricular tutoring, and interest classes [35,36] (Hypothesis 4a), as well as watching TV and surfing online and playing online games [38] (Hypothesis 4b) would have a stronger positive relationship with depressive symptoms among girls than boys. On the other hand, the duration of extracurricular physical exercise [39] would be more strongly related to depressive symptoms in negative manner among girls than boys (Hypothesis 4c).

Question 5: Does family economic status moderate the relationship between extracurricular activities and depressive symptoms among Chinese early adolescents? As educational and interest activities may promote human capital of adolescents experiencing economic disadvantage, we proposed that time spent on homework, extracurricular tutoring, and interest classes [40] (Hypothesis 5a) would have stronger negative relationships with depression in adolescents with a lower economic status. On the other hand, as poorer adolescents typically lack entertainment activities because of economic constraints, watching TV, and surfing online and playing games online, would have stronger relationships with depressive symptoms among students with a higher family economic status relative to students with lower family economic status [41] (Hypothesis 5b). Finally, we proposed that time spent on physical exercise would have a stronger negative relationship with depressive symptoms in students with a lower family economic status relative to students with a higher family economic status [42,43] (Hypothesis 5c).

## 2. Methods

### Participants and Procedure

The research data were derived from the China Education Panel Study (CEPS). The CEPS survey took the seventh and ninth grades of the 2013–2014 school year as the starting point; it was followed up annually thereafter. The CEPS took the average education level of the population and the proportion of the floating population as stratified variables and used the multistage probability proportional to scale (PPS) sampling method to randomly select 112 schools in 28 counties. The CEPS survey collected detailed information from students and their parents, school administrators, class teachers, and teachers. So far, only the data of the 2013–2014 and 2014–2015 school year are open for application from scholars.

In the present study, given that the baseline survey questionnaire and the follow-up questionnaire have different items and scoring methods for participation in extracurricular activities, and the items in the follow-up survey questionnaire are more comprehensive and specific, we used the follow-up data of the 2014–2015 academic year (Grade 8). After we removed the participants without follow-up data or with missing information, there were 9672 students (*M*_age_ = 14.54 years, *SD* = 0.70 years) in the final sample. Among them, there were 4342 girls, 4706 boys, and 624 without gender information. Regarding residence, 4820 students had a rural registered permanent residence, 4392 students had an urban registered permanent residence, and 460 were without related information.

## 3. Measures

### 3.1. Depressive Symptoms

Depressive symptoms were measured by 9 items in the CEPS Student Questionnaire. The items were selected from the Patient Health Questionnaire (PHQ-9) and the Center for Epidemiological Studies-Depression (CES-D). The PHQ-9 is a commonly used depression assessment scale with 9 items [59], and the CES-D scale is a short self-reported scale designed to measure depressive symptomatology in the general population [60]. These items asked participants whether they had the following feelings in the past 7 days: depressed, too depressed to concentrate on doing things, unhappy, bored with life, unable to do things, sad, nervous, overly worried, and having a bad presentiment. All of the items were rated on the following 5-point scale: never = 1, rarely = 2, sometimes = 3, often = 4, always = 5. The average score of these 9 items was used to generate a depressive symptoms score, with a high average score indicating a high level of depressive symptoms. This scale was reported to be reliable and valid in previous studies [61]. The Cronbach’s alpha in this study was 0.92.

### 3.2. After-School Activity Participation

The predictor variable was time spent by students in extracurricular activities on weekdays and weekends. We used question C13 and the five sub-items of questions B7 and B8 from the CEPS Student Questionnaire. Questions B7 and B8 ask participants, “From Monday to Friday, how do you arrange your after-school activities each day?” and “On weekends, how do you arrange your after-school activities each day?” The selected sub-items were “completing homework assigned by teachers”, “attending extracurricular tutoring (related to schoolwork)”, “attending interest classes (unrelated to schoolwork)”, “watching TV”, and “surfing the Internet and playing games”. The answer choices for after-school activities from Monday to Friday were 1 = none, 2 = less than 1 h, 3 = approximately 1–2 h, 4 = approximately 2–3 h, 5 = approximately 3–4 h, and 6 = approximately 4 h or more. The answer choices for weekend after-school activities were 1 = none, 2 = less than 2 h, 3 = approximately 2–4 h, 4 = approximately 4–6 h, 5 = approximately 6–8 h, and 6 = approximately 8 h or more. Question C13 was completed by students and measured the students’ weekly physical exercise time in the following format: usually ___ days per week, ___ minutes per day. The value of this question is converted into natural logarithmic form.

### 3.3. Demographic Measures

First, demographic variables including gender, household registration and only child status were set as dummy variables. For these variables, boys, agricultural household registration, and only children were assigned a value of 1, and the other categories were assigned a value of 0. Second, we also used other demographic variables including family economic status, parents’ highest educational level, and school ranking. Among them, family economic status was measured by questions “What is the current economic condition of your family?” in the parents’ questionnaire and “How do you feel about your family’s financial situation right now?” in the student questionnaire, with a 5-point frequency response scale used in both items: 1 = very difficult, 2 = relatively difficult, 3 = moderate, 4 = relatively wealthy, and 5 = very wealthy. We gave priority to data reported by parents; when the parent-reported data were missing, we filled in data reported by students. The final data were merged into three categories: 1 and 2 = difficult (1), 3 = moderate (2), and 4 and 5 = wealthy (3). As a result, there were 1917 students (19.8%) from families with a difficult economic status, 6734 students (69.6%) from families with a moderate economic status, and 537 students (5.6%) from families with a wealthy economic status, with 484 cases with missing information.

For parents’ highest educational level, the educational levels of the student’s father and mother were compared, and the higher value was taken, with the following categories: 1 = no education, 2 = primary school, 3 = middle school, 4 = technical secondary school/technical school, 5 = vocational high school, 6 = high school, 7 = college, 8 = bachelor’s degree, 9 = graduate and above.

School ranking was reported by the school authority. The ranking of schools in the county (district) was divided into five categories: worst, inferior, middle, upper middle, and best. The five categories were combined into three categories, namely, middle and below, upper middle, and best, which were assigned as values of 1, 2, and 3, respectively. This categorization method was used in previous studies [62].

### 3.4. Data Analytic Strategy

The data in this study involved two levels (i.e., students were nested in schools), with students at the first level and schools at the second level. Considering that the school environment is an important factor affecting student mental health, and that learning in different types of schools may have different impacts on students, it was necessary to test whether it was suitable to adopt the two-level linear model analysis method. The results based on the intercept model show that the intragroup correlation coefficient (ICC) was 0.03 [0.022/(0.022 + 0.704)]. When the ICC does not reach 0.059, there is no group effect, and the multilevel model analysis method is unnecessary [63]. Therefore, we used a multiple regression model to analyze the data, controlling for school rank, family economic status, parents’ highest educational level, student gender, household registration, and only child status. In the multiple regression analyses, Model 1 contained only the controlled variables, and we added all variables of after-school activities in Model 2. Moreover, in order to examine the moderating effects of gender and family economic status on the associations between after-school activities and adolescent depression symptoms, we added the interaction between gender and extracurricular activities, and the interaction between family economic status and extracurricular activities in Model 3 and Model 4, respectively. Simple effect tests were conducted using multiple regression analyses to explore the relationship between extracurricular activity participation and depressive symptoms among adolescents with different gender and with different family economic status, respectively. All analyses were performed using SPSS 21.0 (International Business Machines Corporation, Armonk, NY, USA).

## 4. Results

### 4.1. Gender and Family Economic Status Differences on All Variables

Table 1 shows the means and standard deviations of all variables according to student’s gender (boys and girls) and family economic status (difficult, moderate, and wealthy).

Regarding various extracurricular activities, girls spent more time completing homework assigned by teachers on weekdays and weekends (weekdays: *t*_homework_ = 7.98, *p* < 0.001, Cohen’s *d* = 0.18; weekends: *t*_homework_ = 5.00, *p* < 0.001, Cohen’s *d* = 0.11), attending extracurricular tutoring on weekend (*t*_tutoring_ = 2.48, *p <* 0.05, Cohen’s *d* = 0.06), and attending interest classes on weekends (*t*_interest class_ = 3.27, *p* < 0.001, Cohen’s *d* = 0.06) than did boys, while boys spent more time attending extracurricular tutoring on weekdays (weekdays: *t*_tutoring_ = −2.13, *p <* 0.05, Cohen’s *d* = −0.04), watching TV (weekdays: *t*_watching TV_ = −5.81, *p* < 0.001, Cohen’s *d* =−0.13; weekends: *t*_watching TV_ = −5.96, *p* < 0.001, Cohen’s *d* = −0.13), surfing the Internet (weekdays: *t*_online_ = −9.92, *p* < 0.001, Cohen’s *d* = −0.21; weekends: *t*_online_ = −16.47, *p* < 0.001, Cohen’s *d* = −0.36), and exercising (*t*_exercising_ = −11.31, *p* < 0.001, Cohen’s *d =* −0.25) than did girls. These results supported Hypothesis 1a and 1b, except for the extracurricular tutoring on weekdays. Finally, girls’ depressive symptoms were significantly higher than those of boys (*t* = 4.13, *p* < 0.001, Cohen’s *d* = 0.09). This result supported Hypothesis 1c.

Second, there were significant differences in the time spent in various extracurricular activities among students from families with different economic statuses. Specifically, students from families with “difficult” economic status spent less time completing homework assigned by teachers (weekdays: *M*_difficult_ = 3.37, *M*_moderate_ = 3.54, *M*_wealthy_ = 3.61; weekends: *M*_difficult_ = 2.89, *M*_moderate_ = 3.04, *M*_wealthy_ = 3.06), attending extracurricular tutoring (weekdays: *M*_difficult_ = 1.38, *M*_moderate_ = 1.65, *M*_wealthy_ = 1.80; weekends: *M*_difficult_ = 1.33, *M*_moderate_ = 1.77, *M*_wealthy_ = 1.99), taking interest classes (weekdays: *M*_difficult_ = 1.29, *M*_moderate_ = 1.38, *M*_wealthy_ = 1.59; weekends: *M*_difficult_ = 1.24, *M*_moderate_ = 1.40, *M*_wealthy_ = 1.57), playing games online (weekdays: *M*_difficult_ = 2.11, *M*_moderate_ = 2.25, *M*_wealthy_ = 2.52; weekends: *M*_difficult_ = 2.32, *M*_moderate_ = 2.66, *M*_wealthy_ = 2.93), and physical exercising (*M*_difficult_ = 4.52, *M*_moderate_ = 4.72, *M*_wealthy_ = 4.92) than did students from moderate and wealthy families. On the other hand, students from families with difficult economic status spent more time watching TV (weekdays: *M*_difficult_ = 2.63, *M*_moderate_ = 2.42, *M*_wealthy_ = 2.47; weekends: *M*_difficult_ = 2.83, *M*_moderate_ = 2.74, *M*_wealthy_ = 2.73). These results supported Hypothesis 2a and partially supported Hypothesis 2b (watching TV). Besides, depressive symptoms of students from families with different economic statuses were significantly different (*F* (2, 9185) = 20.37, *p* < 0.001), and depressive symptoms of students from families with a difficult economic status were higher than those of students from families with moderate and wealthy economic statuses (*M*_difficult_ = 2.29, *M*_moderate_ = 2.16, *M*_wealthy_ = 2.09). This result supported Hypothesis 2c. The findings are presented in Table 1.

### 4.2. Predictive Effects of Extracurricular Activities on Depressive Symptoms

Correlation analyses showed that for most extracurricular activities, time spent on the activity was significantly correlated with depressive symptoms (see Table 2). Except for time spent attending extracurricular tutoring classes on weekends and attending interest classes on weekdays and weekends, time spent on the other extracurricular activities was significantly correlated with students’ depressive symptoms. Specifically, time spent on physical exercise was negatively related to depressive symptoms (*r* = −0.065), while time spent on the other extracurricular activities was positively associated with depressive symptoms (*r* = 0.032~0.090), with a low effect size.

The results of Model 1 in Table 3 indicated that girls’ depressive symptoms were higher than those of boys (*β* = −0.041, *p* < 0.001), that family economic status had a significant negative effect on students’ depressive symptoms (*β* = −0.060, *p* < 0.001), and that the depressive symptoms of non-only children were higher than those of only children (*β* = 0.039, *p* = 0.001).

The results of Model 2 show that time spent completing homework assigned by teachers on weekdays and weekends had a positive effect on students’ depressive symptoms (*β* = 0.099, *p* < 0.001; *β* = 0.076, *p* < 0.001, respectively). Time spent attending extracurricular tutoring on weekdays and weekends also had positive effects on students’ depressive symptoms (*β* = 0.031, *p* = 0.009; *β* = 0.025, *p* < 0.05, respectively). In addition, for recreational activities, time spent surfing the Internet and playing games had a positive effect on students’ depressive symptoms (weekdays: *β* = 0.070, *p* < 0.001; weekends: *β* = 0.097, *p* < 0.001), while time spent watching TV did not have an effect on students’ depressive symptoms. Hypothesis 3a was partially supported. Moreover, participating in physical exercise on weekdays and weekends had negative effects on students’ depressive symptoms (*β* = −0.056, *p* < 0.001; *β* = −0.059, *p* < 0.001). Hypothesis 3b was partially supported.

### 4.3. Gender and Family Economic Status as Moderators on the Relationship between Extracurricular Activities and Depressive Symptoms

The results in Table 4 show that the interaction between time spent attending interest classes on weekends and gender on depressive symptoms was significant (*β* = −0.035, *p* < 0.05). Moreover, the interaction of time spent surfing the Internet and playing games with gender was significant (weekdays: *β* = −0.059, *p* < 0.005; weekends: *β* = −0.038, *p* < 0.05). The simple effect test (Table 5) showed that time spent attending interest classes on weekends had a positive effect on girls’ depressive symptoms (*β*_girls_ = 0.034, *p* < 0.05), while the effect was not significant for boys (*β*_boys_ = −0.016, *p* > 0.05). Moreover, time spent playing online games on weekdays and weekends had greater positive effects on depressive symptoms among girls than boys (weekdays: *β*_boys_ < 0.05, *p* = 0.019; *β*_girls_ = 0.108, *p* < 0.001; weekends: *β*_boys_ = 0.079, *p* < 0.001; *β*_girls_ = 0.119, *p* < 0.001). Therefore, while Hypotheses 4a and 4b were partially supported, Hypothesis 4c was not supported.

Model 4 in Table 4 shows the interactive effect between extracurricular activities and family economic status on depressive symptoms; Table 6 shows the simple effect test for the effect of extracurricular activities on depressive symptoms among students from families with different economic statuses. First, results indicated that the interactive effects between time spent completing homework assigned by teachers and family economic status on students’ depressive symptoms were significant (weekdays: *β*_moderate vs. difficult_ = 0.081, *p* < 0.001; *β*_wealthy vs. difficult_ = 0.038, *p* < 0.005; weekends: *β*_moderate vs. difficult_ = 0.071, *p* < 0.005; *β*_wealthy vs. difficult_ = 0.028, *p ≤* 0.05). The simple effect test indicates that the positive effects of time spent completing homework on depressive symptoms among students from moderate and wealthy families were significant (weekdays: *β*_moderate_ = 0.113, *p* < 0.001; *β*_wealthy_ = 0.164, *p* < 0.001; weekends: *β*_moderate_ = 0.091, *p* < 0.001; *β*_wealthy_ = 0.114, *p* < 0.05), while the effects among students from families with difficult economic status were not significant (Weekdays: *β*_difficult_ = 0.032, *p* > 0.05; Weekends: *β*_difficult_ = 0.009, *p* > 0.05). Hypothesis 5a was supported.

Second, the results indicated that the effects of time spent attending extracurricular tutoring on students’ depressive symptoms were moderated by family economic status (weekdays: *β*_wealthy vs. difficult_ = 0.034, *p <* 0.05; *β*_moderate vs. difficult_ = 0.065, *p <* 0.05; weekends: *β*_wealthy vs. difficult_ = 0.032, *p <* 0.05). The results of the simple effect test show that the positive effects of time attending extracurricular tutoring on depressive symptoms among students from moderate and wealthy families were significant (weekdays: *β*_moderate_ = 0.033, *p <* 0.05; *β*_wealthy_ = 0.100, *p <* 0.05; weekends: *β*_wealthy_ = 0.104, *p <* 0.05), while the effects among students from a difficult family economic status were not significant (weekdays: *β*_difficult_ = −0.024, *p* > 0.05; weekends: *β*_difficult_ = 0.005, *p* > 0.05). Hypothesis 5a was supported.

Third, we found that the effects of time spent surfing the Internet and playing games on students’ depressive symptoms were moderated by family economic status (weekdays: *β*_moderate vs. difficult_ = 0.056, *p* < 0.001; weekends: *β*_wealthy vs. difficult_ = 0.033, *p <* 0.05). The simple effect test indicates that time spent surfing the Internet and playing games on weekdays had a greater positive effect on depressive symptoms among students from moderate families than among students from families with a difficult economic status (*β*_moderate_ = 0.055, *p* < 0.001; *β*_difficult_ = 0.092, *p <* 0.005) and that time spent surfing the Internet and playing games on weekends had a greater positive effect on depressive symptoms among students from wealthy families than among students from families with a difficult economic status (*β*_wealthy_ = 0.254, *p* < 0.001; *β*_difficult_ = 0.100, *p <* 0.001). Therefore, while Hypotheses 5a and 5b was partially supported, Hypothesis 5c was not supported. In summary, we present the degree of support for the different hypotheses in Table 7.

## 5. Discussion

Generally speaking, the present findings showed that participation in extracurricular activities such as study, leisure, interest, and exercise were associated with depressive symptoms among Chinese early adolescents and that the relationships were moderated by gender and family economic status. As no related findings have been reported in different Chinese contexts, the present study is a pioneer. However, it should be noted that the effect size of the significant findings is low.

### 5.1. Extracurricular Activities and Depressive Symptoms among Early Adolescents

We found that time spent writing homework assigned by teachers, attending extracurricular tutoring classes, and playing online games were positively associated with students’ depressive symptoms. This observation is consistent with the previous studies [18,19,20,21,22,23]. Besides, consistent with other studies [28,29,30], time spent taking part in physical exercise was negatively associated with students’ depressive symptoms.

We explain the above observations in terms of the socio-cultural attributes of China. Influenced by traditional Confucian values, Chinese parents attach great importance to their children’s educational achievements and regard these achievements as bringing glory to the whole family [64]. Hence, parents and schoolteachers would have excessive academic requirements for teenagers, such as asking them to participate in many remedial classes. Unfortunately, excessive time spent in inactive learning may lead to excessive learning pressure, which may in turn lead to depressive symptoms among adolescents [23].

Moreover, although the effectiveness of physical exercise in improving an individual’s emotional state has been supported by many Western studies [28,30], few studies have been conducted in China. When people take part in physical exercise, their attention is diverted, which releases psychological pressure, regulates nervous emotions, and improves negative emotions such as anxiety and irritability. Additionally, physical exercise can improve the functions of various organs and increase the endurance of the heart and lungs. Improving physical health to promote mental health is an important way that physical exercise has psychological effects [65,66]. Unfortunately, Chinese parents may hold the traditional belief that too much “play” involving physical activities is non-beneficial to the development of children. Finally, many studies showed that adolescents’ addiction to the Internet contributed to psychological maladjustment [23].

The positive association of time spent surfing the Internet and playing online games with depressive symptoms was greater for girls than boys, which is consistent with most previous study results [38]. The reason for this gender difference may be due to the difference in self-efficacy. Studies have pointed out that men have always play a dominant role in the development of computers and the Internet and that they have a strong spirit of exploration and better technological skills to use the Internet. As a result, boys can gain more self-efficacy in the use of the Internet. Moreover, the emergence of online games has allowed men to gain a higher sense of accomplishment and experience more self-worth via the virtual environment [51,52,53]. However, girls are more sensitive to the negative impact of excessive use of the Internet because society does not advocate excessive use of the Internet [67]. Girl’s stronger recognition of social norms makes them more likely to experience self-blame as they surf the Internet, which leads to a decrease in happiness and an increase in negative experiences such as depressive symptoms. In addition, we have found that time spent attending interest classes on weekends would be more strongly related to the depressive symptoms among girls than boys in a positive manner, which is inconsistent with previous studies [33]. Yu [68] argued that, in China, the first consideration for many interest classes was parents’ needs. Some parents made decisions for their children according to their own expectations of their children’s growth. Typically, those “interests” that can improve academic performance and contribute to career achievement are often quite popular (such as foreign languages). Obviously, to a large extent, interest classes are constructed with reference to the adult world, not children’s real interests and preferences. If joining the interest class is not the children’s own choice, it may lead to their resentment and have negative consequences. Such negative effect is more intense among girls probably because girls in fact spend more time in interest classes than boys on weekends, which may make them feel tired and burdensome. Obviously, future research should investigate whether adolescents choose to join the interest classes in a voluntary manner would have any effect on their well-being.

Besides gender, family economic status was also found to be a moderating factor. Time spent completing homework assigned by teachers on weekdays and weekends and time spent attending extracurricular tutoring classes on weekdays were positively associated with depressive symptoms among students from financially moderate families and wealthy families, while no similar effect was found in students from families with economic difficulty, thus supporting Hypothesis 5a. This result can be explained by the conjecture that parents with higher family economic status tend to value their children’s academic achievements, and they may “orchestrate” their children to join academic activities with stronger supervision [40,69]. Greater parental participation and academic supervision implies higher academic expectations and requirements for children, as well as greater competition and psychological stress for adolescents, which may further lead to an increase in adolescent depressive symptoms. On the other hand, lower class children might benefit from homework, extra-curricular tutoring, and interest classes because the related activities would promote human capital helping them to move up the social ladder.

Moreover, we found that time spent surfing the Internet and playing games had a stronger positive relationship with depressive symptoms among students from financially moderate and wealthy families than among students from families with a difficult economic status, thus providing support for Hypothesis 5b. This observation may be due to the fact that teenagers with high family economic status have more material possessions (e.g., computers), and their families are more likely to provide them with the opportunity and time to surf the Internet without much parental supervision at their free time [41]. As a result, such adolescents would suffer from depressive symptoms when spending excessive time playing online games. However, for children with low family economic status, surfing Internet and playing online games may simply be entertainment because they lack entertainment due to financial constraint.

### 5.2. Strengths and Limitations

This study makes important contributions to the literature by systematically examining the associations between participation in after-school activities and early adolescents’ depressive symptoms and further investigating the moderating effects of adolescents’ gender and family economic status. First, as predicted, we found that there are gender as well as socio-economic differences in early adolescents’ participation in extra-curricular activities and depressive symptoms. Although some of the findings are “commonly known”, they are still important because no study has been conducted in China. Second, the present study found that participation in different after-school extracurricular activities were differentially associated with depressive symptoms among early adolescents in China. Third, we found that gender and socio-economic status are moderators of the relationships between participation in extra-curricular activities and depression. Specifically, gender moderated the relationships on some measures of extra-curricular activities. Besides, while excessive extracurricular academic activities and playing games online are detrimental to adolescent mental health, especially among students from moderate and wealthy families, physical exercise is beneficial to adolescent mental health in all participants. Theoretically, the present study expands our understanding of the relationship between participation in extra-curricular activities and adolescent depression and the related moderators, which is not well-articulated in the existing scientific literature. Besides, this pioneer study contributes to the Chinese scientific literature. As pointed out by Oberle et al. [70], as “many of the previous studies in this field have been conducted in the context of the United States” (p. 13), the present study in the Chinese context is an important contribution to the field.

Although we obtained some novel findings, there are some limitations in this study. First, this study adopted a cross-sectional survey design, which is not as powerful as longitudinal designs. In this study, we were unable to adequately test the causes and effects of after-school extracurricular activities and depression symptoms because both variables occurred at the same time. Future research should consider adopting longitudinal research designs to examine the predictive relationships between participation in after-school extracurricular activities and adolescents’ depressive symptoms. Second, the China Education Panel Survey collected data from students by a self-reported questionnaire, which may cause response bias. However, this approach is commonly used to understand adolescent behavior in the field. Obviously, future research should gather information from adolescents, parents, and teachers. Third, in the current study, after-school extracurricular activities and depressive symptoms were measured by using self-reported questionnaires from the CEPS. However, it is noteworthy that self-reported rating scales, such as the Centre for Epidemiologic Studies Depression Scale (CES-D), have been commonly used to assess depression [71,72]. Fourth, it should be noted that different motivation for participating in after-school activities will influence the feedback of the activities, such as happiness, enjoyment, depression, and frustration [73]. While activities of surfing the Internet and attending interest classes may be “entertainment”, they are also activities for promoting human capital in the future. As such, the motivation for participating in after-school extracurricular activities and individual adolescents’ related variability should be considered in future studies. Fifth, other factors related to depressive symptoms were not taken into account, such as negative automatic thoughts, learning achievement, health status, interpersonal relations, and school types [74]. Future research should include these factors where appropriate. Finally, as our findings showed that the magnitude of the effect was low, the significant results may simply be due to large sample. As there is a distinction between theoretical significance and practical significance, we should be cautious in interpreting these results. However, as argued by Orberle et al. [70], studies in this field commonly generated findings with a small effect size. Besides, they pointed out that “even small effect sizes indicating the positive links between ECAs (extracurricular activities) and positive outcomes are important because effects are cumulative in nature of development where small changes compound over contexts and time” (p. 12).

### 5.3. Practical Implications

First, our results showed that excessive time spent on extracurricular tutoring was associated with an increase in depression, and parents should re-think about the role of tutoring to avoid unnecessary and compulsory tutoring and respect students’ independent choices. Second, as addiction to TV or the Internet was associated with depressive symptoms, schools, parents, and society must strengthen education and guidance, create conditions for students to participate in suitable leisure activities, and prevent them from over-engaging in extracurricular activities in the virtual world. Third, schools, parents, and adolescents should recognize the benefits of appropriate physical exercise for early adolescents’ mental health, which could reduce the academic burden of adolescents. Finally, we should also take gender and family economic conditions into account when designing youth enhancement programs for families with early adolescents. For example, we found that the positive associations between time spent playing online games and depressive symptoms were stronger among girls than boys, parents should pay more attention to the Internet use and emotional problems of adolescent girls and adopt emotionally warm education in the process of parental guidance. Additionally, parents of financially better families also need to arrange their children’s after-school life with care and supervise their children’s online entertainment time to avoid the negative affect on their children’ s mental health. In short, as argued by Oberle et al. [70], examination of the relationship between extra-curricular activities and adolescent well-being is “critical because it contributes to identifying ways for promoting the positive development of the whole child with a specific focus on extracurricular programs that take place in the community”(p.12). Besides, arranging appropriate extra-curricular activities for students is in line with the holistic model of child development [75]. Most importantly, extra-curricular activities may play an important role to promote the psychological well-being of students under COVID-19 [76,77].

## 6. Conclusions

Overall, our findings suggested that (1) there were gender and class differences in participation in extra-curricular activities; (2) time spent completing homework assigned by teachers, attending extracurricular tutoring, and playing online games were positively associated with students’ depressive symptoms, while time spent participating in physical exercise were negatively associated with students’ depressive symptoms; and (3) the associations of extracurricular activities with students’ depressive symptoms were moderated by gender and family economic status. In particular, the positive associations between time spent playing online games on weekdays and weekends and depressive symptoms were stronger among girls than boys. Time spent completing homework and playing online games were more positively associated with depressive symptoms among students from moderate and wealthy families than among students from families with a relatively poorer economic status.

## Figures and Tables

**Table 1:** Means and standard deviations of all variables by gender and family economic status.

Variable	*M (SD)*		*t*	*M (SD)*			*F*	Tamhane Post Hoc
	Girls	Boys		Difficult	Moderate	Wealthy		
**Dependent variable**								
Depression	2.22 (0.81)	2.15 (0.89)	4.13 ***	2.29 (0.82)	2.16 (0.85)	2.09 (0.91)	20.37 ***	1 > 2, 3
**Independent variables** **(weekdays)**								
Completing homework assigned by teachers	3.62 (1.10)	3.42 (1.19)	7.98 ***	3.37 (1.22)	3.54 (1.13)	3.61 (1.13)	18.70 ***	1 < 2, 3
Attending extracurricular tutoring	1.58 (1.21)	1.63 (1.27)	−2.13 *	1.38 (0.98)	1.65 (1.28)	1.80 (1.42)	43.72 ***	1 < 2, 3
Attending interest classes	1.38 (0.91)	1.37 (0.93)	0.43	1.29 (0.82)	1.38 (0.92)	1.59 (1.14)	23.83 ***	1 < 2, 3; 2 < 3
Watching TV	2.37 (1.34)	2.55 (1.45)	−5.81 ***	2.63 (1.47)	2.42 (1.37)	2.47 (1.50)	16.19 ***	2 < 1
Surfing the Internet and playing games	2.09 (1.26)	2.38 (1.56)	−9.92 ***	2.11 (1.45)	2.25 (1.41)	2.52 (1.57)	16.88 ***	1 < 2, 3; 2 < 3
**(weekends)**								
Completing homework assigned by teachers	3.07 (0.97)	2.96 (1.09)	5.00 ***	2.89 (1.08)	3.04 (1.02)	3.06 (1.02)	15.96 ***	1 < 2, 3
Attending extracurricular tutoring	1.73 (1.16)	1.66 (1.15)	2.48 *	1.33 (0.83)	1.77 (1.19)	1.99 (1.33)	132.33 ***	1 < 2, 3; 2 < 3
Attending interest classes	1.40 (0.83)	1.35 (0.82)	3.27 ***	1.24 (0.69)	1.40 (0.83)	1.57 (0.96)	46.01 ***	1 < 2, 3; 2 < 3
Watching TV	2.68 (1.11)	2.83 (1.29)	−5.96 ***	2.83 (1.23)	2.74 (1.19)	2.73 (1.26)	4.25 *	2 < 1
Surfing the Internet and playing games	2.37 (1.18)	2.83 (1.45)	−16.47 ***	2.32(1.37)	2.66 (1.32)	2.93 (1.44)	63.60 ***	1 < 2, 3; 2 < 3
Engaging in physical exercise	4.58 (0.81)	4.80 (0.99)	−11.31 ***	4.52 (0.91)	4.72 (0.91)	4.92 (0.89)	51.42 ***	1 < 2, 3; 2 < 3
**Control variables**								
Gender	-	-	-	0.55 (0.50)	0.51 (0.50)	0.56 (0.50)	8.13 ***	2 < 1, 3
Household registration	0.51 (0.50)	0.53 (0.50)	−1.67	0.70 (0.46)	0.48 (0.50)	0.39 (0.49)	171.64 ***	3 < 1, 2; 2 < 1
Family economic status	1.86 (0.48)	1.84 (0.51)	1.57	-	-	-	-	-
Only child status	1.58 (0.49)	1.52 (0.50)	5.11 ***	1.75 (0.43)	1.50 (0.50)	1.48 (0.50)	202.80 ***	2 < 1; 3 < 1
Parents’ highest educational level	4.65 (2.04)	4.60 (2.02)	1.35	3.71 (1.61)	4.78 (2.04)	5.71 (2.13)	312.90 ***	1 < 2, 3; 2 < 3
School ranking	2.05 (0.63)	2.03 (0.65)	2.02 *	1.86 (0.60)	2.07 (0.64)	2.19 (0.65)	99.99 ***	1 < 2, 3; 2 < 3

Note: * *p* < 0.05, *** *p* < 0.001. Standard deviations are in parentheses. Bold is used to distinguish dependent variable (depression), independent variables (extracurricular activities on weekdays and weekends), and control variables.

**Table 2:** Correlation between extracurricular activities and depressive symptoms.

	Depression
**Weekdays**	
Completing homework assigned by teachers	0.090 ***
Attending extracurricular tutoring	0.039 ***
Attending interest classes	0.018
Watching TV	0.032 **
Surfing the Internet and playing games	0.062 ***
**Weekends**	
Completing homework assigned by teachers	0.064 ***
Attending extracurricular tutoring	0.005
Attending interest classes	0.009
Watching TV	0.035 ***
Surfing the Internet and playing games	0.074 ***
Engaging in physical exercise	−0.065 ***
**Control variables**	
Gender	−0.043 ***
Household registration	0.026 *
Only child status	−0.065 ***
Parents’ highest educational level	0.055 ***
Family economic status	−0.033 **
School ranking	0.008

Note: * *p* < 0.05, ** *p* < 0.01, *** *p* < 0.001. Bold is used to distinguish independent variables (extracurricular activities on weekdays and weekends) and control variables.

**Table 3:** Effects of extracurricular activities on depressive symptoms.

	Model 1	Model 2a	Model 2b
	β	β (Weekdays)	β (Weekends)
**Control variables**			
Gender	−0.041 ***	−0.029 **	−0.040 ***
Household registration	0.004	0.009	0.011
Only child status	0.039 **	0.042 **	0.044 ***
Parents’ highest educational level	−0.005	−0.011	−0.014
Family economic status	−0.060 ***	−0.060 ***	−0.066 ***
School ranking (upper middle)	−0.025	−0.022	−0.021
School ranking (best)	−0.007	−0.004	−0.003
**Independent variables**			
Completing homework assigned by teachers		0.099 ***	0.076 ***
Attending extracurricular tutoring		0.031 **	0.025 *
Attending interest classes		−0.004	0.007
Watching TV		0.001	0.012
Surfing the Internet and playing games		0.070 ***	0.097 ***
Engaging in physical exercise		−0.056 ***	−0.059 ***
Sample size	9672		

Note: * *p* < 0.05, ** *p* < 0.01, *** *p* < 0.001. Two dummy variables were generated for school ranking: upper middle vs. medium and below, and best vs. medium and below. The reference group is medium and below. Bold is used to distinguish independent variables (extracurricular activities) and control variables.

**Table 4:** Moderating effects of gender and family economic status on the effects of extracurricular activities on depressive symptoms.

	Model 3a	Model 3b	Model 4a	Model 4b
	β (Weekdays)	β (Weekends)	β (Weekdays)	β (Weekends)
**Control variables**	Controlled	Controlled	Controlled	Controlled
**Independent variables**				
Completing homework assigned by teachers	0.102 ***	0.083 ***	0.022	0.009
Attending extracurricular tutoring	0.009	0.018	−0.037	−0.039
Attending interest classes	0.013	0.034 *	0.032	0.041
Watching TV	−0.026	0.013	0.011	0.042
Surfing the Internet and playing games	0.113 ***	0.121 ***	0.032 *	0.079 ***
Engaging in physical exercise	−0.079 ***	−0.085 ***	−0.047	−0.051
Completing homework assigned by teachers * gender	−0.002	−0.006	-	-
Attending extracurricular tutoring * gender	0.029	0.012	-	-
Attending interest classes * gender	−0.024	−0.035 *	-	-
Watching TV * gender	0.033	−0.003	-	-
Surfing the Internet and playing games * gender	−0.059 **	−0.038 *	-	-
Physical exercise * gender	0.025	0.026	-	-
Extracurricular activities * family economic status				
Completing homework assigned by teachers * (moderate vs. difficult)			0.081 ***	0.071 **
Attending extracurricular tutoring * (moderate vs. difficult)			0.065*	0.054
Attending interest classes * (moderate vs. difficult)			−0.032	−0.036
Watching TV * (moderate vs. difficult)			0.007	−0.030
Surfing the Internet and playing games * (moderate vs. difficult)			0.056 ***	0.005
Engaging in physical exercise * (moderate vs. difficult)			−0.008	−0.006
Completing homework assigned by teachers * (wealthy vs. difficult)			0.038 **	0.028 *
Attending extracurricular tutoring * (wealthy vs. difficult)			0.034 *	0.032 *
Attending interest classes * (wealthy vs. difficult)			−0.020	−0.004
Watching TV * (wealthy vs. difficult)			−0.014	−0.014
Surfing the Internet and playing games * (wealthy vs. difficult)			0.027	0.033 *
Engaging in physical exercise * (wealthy vs. difficult)			−0.018	−0.022
Sample size	9672		9672	

Note: * *p* < 0.05, ** *p* < 0.01, *** *p* < 0.001. Two dummy variables were generated for family economic status: moderate vs. difficult and wealthy vs. difficult. The reference group is the difficult group. Bold is used to distinguish independent variables (extracurricular activities) and control variables.

**Table 5:** Effects of extracurricular activities on depressive symptoms among boys and girls.

	Weekdays		Weekends	
	β (Boys)	β (Girls)	β (Boys)	β (Girls)
**Control variables**				
Household registration	0.005	0.018	0.003	0.021
Family economic status	−0.072 ***	−0.047 **	−0.081 ***	−0.048 **
Only child status	0.022	0.068 ***	0.023	0.072 ***
Parents’ highest educational level	−0.020	0.001	−0.023	−0.004
School ranking (upper middle)	−0.017	−0.026	−0.021	−0.021
School ranking (best)	0.009	−0.014	0.005	−0.011
**Independent variables**				
Completing homework assigned by teachers	0.096 ***	0.099 ***	0.073 ***	0.079 ***
Attending extracurricular tutoring	0.049 **	0.010	0.032	0.019
Attending interest classes	−0.019	0.012	−0.016	0.034 *
Watching TV	0.020	−0.023	0.011	0.015
Surfing the Internet and playing games	0.041 *	0.108 ***	0.079 ***	0.119 ***
Engaging in physical exercise	−0.045 **	−0.069 ***	−0.047 ***	−0.072 ***

Note: * *p* < 0.05, ** *p* < 0.01, *** *p* < 0.001. Bold is used to distinguish independent variables (extracurricular activities) and control variables.

**Table 6:** Effects of extracurricular activities on depressive symptoms in different family economic status groups.

	Weekdays			Weekends		
	β (Difficult)	β (Moderate)	β (Wealthy)	β (Difficult)	β (Moderate)	β (Wealthy)
**Control variables**						
Gender	−0.035	−0.024	−0.084	−0.035	−0.036 **	−0.133 **
Household registration	−0.012	0.012	0.059	−0.015	0.014	0.059
Only child status	−0.011	0.051 ***	0.084	−0.003	0.049 **	0.100 *
Parents’ highest educational level	−0.034	−0.004	0.014	−0.043	−0.006	0.014
School ranking (upper middle)	−0.066	−0.014	−0.012	−0.052	−0.017	−0.032
School ranking (best)	−0.046	0.004	−0.002	−0.041	0.005	−0.017
**Independent variables**						
Completing homework assigned by teachers	0.032	0.113 ***	0.164 ***	0.009	0.091 ***	0.114 *
Attending extracurricular tutoring	−0.024	0.033 *	0.100*	0.005	0.019	0.104 *
Attending interest classes	0.024	−0.007	−0.028	0.036	−0.002	0.036
Watching TV	0.034	0.000	−0.058	0.039	0.009	−0.031
Surfing the Internet and playing games	0.092 **	0.055 ***	0.156 **	0.100 ***	0.082 ***	0.254 ***
Engaging in physical exercise	−0.037	−0.056 ***	−0.080	−0.047	−0.058 ***	−0.090 *

Note: * *p* < 0.05, ** *p* < 0.01, *** *p* < 0.001. Bold is used to distinguish independent variables (extracurricular activities) and control variables.

**Table 7:** Summarizing the support for the different hypotheses.

Hypotheses	Support or Not
**Hypothesis 1a:** *girls would spend more time on homework, extracurricular tutoring, and interest classes.*	Support
**Hypothesis 1b:** *boys would spend more time on watching TV, surfing online and playing online games, and physical exercise.*	Support
**Hypothesis 1c:** *girls would have greater depressive symptoms compared with boys.*	Support
**Hypothesis 2a:** *time spent on homework, extracurricular tutoring, interest classes, and physical exercise would be more among students with high family economic status.*	Support
**Hypothesis 2b:** *time spent on watching TV and surfing online and playing online games would be more among students with lower family economic status.*	Partial support (watching TV)
**Hypothesis 2c:** *students with lower family economic status would have greater depressive symptoms relative to students with higher family economic status.*	Support
**Hypothesis 3a:** *time spent on homework, extracurricular tutoring, watching TV, and surfing the Internet and playing games would be positively associated with depressive symptoms.*	Partial support (homework, extracurricular tutoring, surfing the Internet and playing games)
**Hypothesis 3b:** *time spent on interest classes and physical exercise would be negatively associated with depressive symptoms.*	Partial support (physical exercise)
**Hypothesis 4a:** *time spent on homework, extracurricular tutoring, and interest classes would be more strongly related to the depressive symptoms among girls than boys in a positive manner.*	Partial support (interest classes)
**Hypothesis 4b:** *time spent on watching TV and surfing online and playing online games would be more strongly associated with depressive symptoms in girls than boys in a positive manner.*	Partial support (surfing online and playing online games)
**Hypothesis 4c:** *negative relationship between the duration of extracurricular physical exercise and depressive symptoms would be stronger in girls than in boys.*	No support
**Hypothesis 5a:** *relative to adolescents with a low family economic status, time spent on homework, extracurricular tutoring, and interest classes would have stronger positive relationship with depressive symptoms among students with high family economic status.*	Partial support (homework, extracurricular tutoring)
**Hypothesis 5b:** *time spent on watching TV and playing games online would have a stronger positive relationship with depressive symptoms among students with a high family economic status than did students with a low family economic status.*	Partial support (surfing the Internet and playing games)
**Hypothesis 5c:** *time spent on physical exercise would have a stronger negative relationship with depressive symptoms among students with a low family economic status than did students with a high family economic status.*	No support

## Data Availability

The datasets generated during and/or analyzed during the current study are available from the first author on reasonable request.
